# Ultrasound-guided 5% dextrose perineural therapy at the axillary nerve for the treatment of shoulder pain in patients with rotator cuff tendinopathy: a randomized, double-blind, sham-controlled trial

**DOI:** 10.2478/abm-2026-0014

**Published:** 2026-04-30

**Authors:** Timporn Vitoonpong, Suda Pipatbannakij, Sopatip Rerkmoung, Chernkhuan Stonsaovapak, Natthiya Tantisiriwat

**Affiliations:** 1 Department of Rehabilitation Medicine, Faculty of Medicine, Chulalongkorn University, Bangkok 10330, Thailand Chernkhuan.S@chula.ac.th; 2 Thai Red Cross Rehabilitation Center, Thai Red Cross Society, Bangkok 10330, Thailand; 3 Department of Rehabilitation Medicine, King Chulalongkorn Memorial Hospital, Thai Red Cross Society, Bangkok 10330, Thailand

**Keywords:** analgesia, dextrose, nerve block, prolotherapy, shoulder pain

## Abstract

**Background:**

5% dextrose perineural injection therapy (PIT) is a low-cost, accessible, and non-toxic pain relief treatment. No study examined the effects of PIT using a 5% dextrose solution for axillary nerve-related shoulder pain.

**Objectives:**

We aimed to investigate the effects of 5% dextrose PIT in the proximal part of the axillary nerve (pAxN) and distal part of the axillary nerve (dAxN) compared to a sham injection, combined with self-exercise in patients with rotator cuff tendinopathy.

**Methods:**

This randomized controlled trial included 40 patients with moderate to severe pain from rotator cuff tendinopathy. Participants were randomly assigned to one of 3 groups: 5% dextrose PIT in pAxN, dAxN, or a sham injection. All groups performed self-exercise at home. Pain was measured using the visual analog scale (VAS), and secondary outcomes included the oxford shoulder score (OSS) and range of motion (ROM). Blinding was maintained for participants and assessors.

**Results:**

After excluding one participant, 39 individuals (13 per group) were included in the analysis. The dAxN group showed significant pain reduction at 2 and 4-weeks, with estimated mean VAS reductions of −2.46 (−3.87, −1.05) and −1.49 (−2.89, −0.08), respectively, compared to the sham group. However, the pAxN group did not demonstrate a significant difference in pain reduction compared to the sham group. There were no significant differences between groups in other outcomes.

**Conclusions:**

Combining 5% dextrose PIT at the dAxN region with self-exercise led to superior short-term pain reduction in patients with rotator cuff tendinopathy. Nonetheless, further large-scale clinical trials are necessary to validate these findings.

Rotator cuff tendinopathy can be managed through various strategies, including exercise, physical therapy, and injections [1, 2]. Exercise effectively reduces pain, improves range of motion (ROM), and enhances upper limb function [1, 2]. However, severe pain may require oral medications or interventions [3]. Long-term use of painkillers can harm kidney or liver function, while corticosteroids have been linked to reduced tendon and articular cartilage integrity [4, 5].

Prolotherapy with hypertonic dextrose is recognized as a regenerative treatment for chronic musculoskeletal pain [6]. Studies demonstrate superior pain relief with intra-tendinous dextrose injections compared to physical therapy or saline injections [7, 8]. However, its superiority over corticosteroids or platelet-rich plasma injections remains inconsistent [9].

5% dextrose perineural injection therapy (PIT) is a lowcost, accessible, and non-toxic pain relief treatment [10]. The mechanism is not fully understood, but potential mechanisms include reduced activation of pain-related substances, hyperpolarization, and modulation of ion channels [11]. PIT using hypertonic dextrose has been studied for pain relief in peripheral nerve entrapments, particularly the median and ulnar nerves [10, 12]. A previous systematic review reported non-serious side effects, including pain and swelling at the injection site after PIT. Additionally, using steroids as the injected substance can cause skin depigmentation at the injection site [10].

The axillary nerve supplies the deltoid and teres minor muscles and provides sensation in various shoulder areas, including the glenohumeral joint, the subacromial bursa, the bicipital groove, and the skin on the posterolateral aspect of the shoulder [3, 13]. Many studies have explored the role of peripheral nerve blocks, including axillary and suprascapular nerve blocks, using anesthetic agents for postoperative pain control [14-16]. Axillary nerve block in surgical conditions is usually performed at the proximal part, such as the inter-scalene or supraclavicular brachial plexus region [17]. Peripheral nerve stimulation has also been reported as an alternative technique for pain reduction, targeting the axillary and suprascapular nerves in cases of chronic rotator cuff tendinopathy that have failed conservative treatment [18]. Previous studies have proposed techniques for ultrasound-guided axillary nerve blocks [3, 13]. Rothe et al. [13] introduced ultrasound-guided injections targeting the proximal part of the axillary nerve (pAxN) in the quadrangular space, while Chang et al. [3] described injection techniques targeting the more distal part of the axillary nerve (dAxN) at the superior lateral humerus.

Walsh et al. [19] conducted a retrospective study of 242 cases that received ultrasound-guided PIT. Among these, 2 cases of shoulder pain following recurrent anterior shoulder dislocation underwent PIT using dexamethasone without an anesthetic agent, with a repeat injection administered 1 month after the first. However, they did not report in terms of efficiency. Despite the existing research on PIT and the axillary nerve’s role in shoulder pain, no studies have examined the effects of PIT using a 5% dextrose solution targeting the axillary nerve in the shoulder pain condition. It is important to note that individuals with musculoskeletal disorders, particularly rotator cuff tendinopathy, may experience neuropathic pain in addition to nociceptive pain [20].

At our institute, PIT of AxN has been used in conjunction with exercise for pain relief in cases of shoulder and arm pain, with rotator cuff tendinopathy being the most common condition. We typically use a 5% dextrose PIT at dAxN or pAxN, with a total volume of 4 mL. The follow-up period is approximately 2-4 weeks after injection. In our experience, these 2 techniques effectively reduce pain, enabling more effective self-exercise with no serious adverse events. To confirm our hypothesis, this study aimed to assess the effectiveness of 5% dextrose PIT at dAxN and pAxN, both combined with self-exercise, compared to a sham injection combined with self-exercise in improving pain levels, ROM, and shoulder function in patients with rotator cuff tendinopathy.

## Methods

This study was a 3-arm, parallel-group, randomized, double-blinded, sham-controlled trial conducted with the permission of the ethics committee of the Faculty of Medicine, Chulalongkorn University (IRB no.464/63). This trial was prospectively registered in the Clinical Trials Registry (TCTR20200805002, released date August 5, 2020).

### Participants

Patients with rotator cuff tendinopathy and shoulder-arm pain were enrolled from outpatient rehabilitation based on these criteria: age 18-60 years, visual analog scale (VAS) pain scores over 4 cm for at least 4 weeks of pain, poor response to prior conservative treatments, and informed consent. We selected patients with moderate to severe pain that interfered with selfexercise requiring intervention to relieve pain. Exclusions were massive/full-thickness cuff tear, recent shoulder interventions within the past month, conditions affecting results (e.g., cervical radiculopathy, brachial plexopathy, etc.), skin issues at injection sites, unexplained weakness/impaired sensation, bleeding tendency, connective tissue diseases, or pregnancy. All participants had a confirmed diagnosis of rotator cuff tendinopathy based on clinical diagnosis combined with imaging studies, either MRI or ultrasound, to exclude massive or full-thickness cuff tears.

### Randomization and allocation concealment

The random allocation sequence was generated using a block of 6 randomization techniques conducted by an independent author (Chernkhuan Stonsaovapak) not involved in the intervention or assessment. Sequential numbers were placed in sealed envelopes and disclosed before administering the intervention.

### Interventions

Participants received education on symptom-avoidance, shoulder ROM exercises, and isotonic strengthening exercises. Lidocaine Hydrochloride jelly 2% was applied to the affected shoulder skin for 30 min at the quadrangular space and superior lateral humeral level. Pinprick sensation was tested before injection to confirm numbness. An author (Suda Pipat-bannakij) followed the instructions without involvement in allocation and intervention. The intervention was administered by Timporn Vitoonpong, who has experience in ultrasound-guided injections for 3 years, using LOGIQ S7 US with a linear transducer and a 3-12 MHz probe.

### 5% Dextrose PIT (proximal and distal part of axillary nerves)

**Figure 1.**
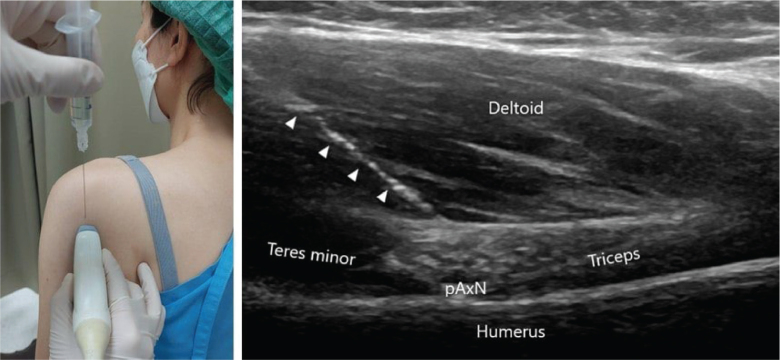
Ultrasound-guided dextrose PIT at quadrangular space (pAxN) white arrowhead, needle; pAxN, proximal part of the axillary nerve.

**Figure 2.**
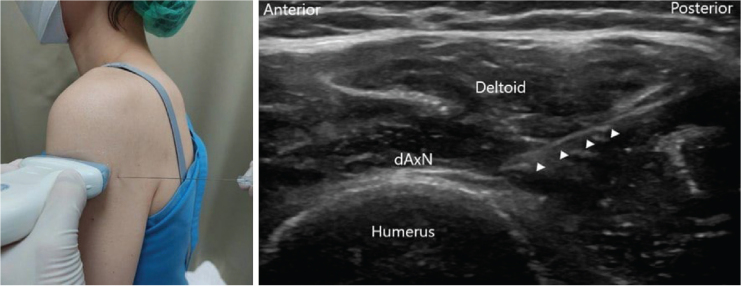
Ultrasound-guided injection at the superior lateral humeral level (dAxN) white arrowhead, needle; dAxN, distal part of the axillary nerve.

Participants received ultrasound-guided injections with 5% dextrose solution, totaling 4 mL (0.4 mL 50% dextrose + 3.6 mL isotonic saline). For pAxN (**Figure 1**), the 25 G spinal needle is injected at the quadrangular space; for dAxN (**Figure 2**), the same needle is at the superior lateral humeral level. To maintain blinding, participants were also subjected to pressure using a blunted tip needle in a separate area: the superior lateral humeral level in the pAxN group and the quadrangular space in the dAxN group [21].

### Sham group

To ensure participant blinding, A blunt-tip needle was applied on the skin at 2 spots, mimicking the positions of the 5% dextrose PIT groups [21].

During the procedure, participants were unable to see the ultrasound monitor, and the assessor (Suda Pipatbannakij) covered needle sites with bandages for effective blinding. If severe shoulder pain occurred later, participants could take paracetamol, note, and document it themselves.

### Outcomes assessment

The primary outcome assessed was shoulder pain, evaluated using the VAS during movement, with the primary endpoint set at 2 weeks after the injection.

The secondary outcomes comprised: The oxford shoulder score (OSS) is a patient-reported questionnaire consisting of 12 items that assess shoulder pain and function experienced in the last 4 weeks. Participants rate their responses using a 5-point Likert scale (1-5), with higher OSS scores indicating greater disability [22]. The shoulder’s active ROM was measured using a goniometer while participants were in a supine position. For forward flexion ROM, the starting position was the anatomical position with the axis at the lateral part of the acromial process. The stationary arm was parallel to the trunk midline, and the moving arm pointed to the lateral humeral epicondyle. During abduction ROM assessment, the starting position was also the anatomical position, with the axis at the anterior aspect of the acromion. The stationary arm remained parallel to the sternum midline, and the moving arm pointed to the lateral humeral epicondyle. For internal and external ROM, participants began with the shoulder abducted at 90°, elbow flexed at 90°, and forearm in supination. The axis was placed at the ulnar side of the olecranon process, the stationary arm was perpendicular to the floor, and the moving arm pointed to the ulnar styloid process [23].

During the initial assessment, we collected demographic data and measured variables such as the VAS, OSS, and ROM of the affected shoulder. Since we aimed to evaluate the shortterm efficacy of the intervention in controlling pain and facilitating self-exercise, follow-up assessments for VAS and ROM were conducted immediately, as well as at 2 and 4 weeks after the injection. For functional outcomes, OSS was reassessed only at 4 weeks. Participants also recorded their pill consumption during follow-up.

### Sample size calculation

As no prior clinical trials were investigating the impact of 5% dextrose PIT on the axillary nerve in patients with rotator cuff tendinopathy, we utilized G*Power to determine the necessary sample size. With a Cohen medium effect size of 0.25, β = 0.1, and α = 0.05, a total of 39 participants (13 per group) were deemed necessary for the study.

### Statistical analysis

In this study, the Stata software version 17 was used. Normality was tested with the Shapiro-Wilk test and histogram. Continuous data were presented as mean and standard deviation. Categorical data were presented as numbers and percentages. We used a mixed model with an interaction for treatment group and time, and a random intercept for participant. This model was compared to a model with a random intercept and slope and found to be superior based on a lower Akaike’s information criterion (AIC) value with VAS and ROM outcome variables. We assessed the changes over time in each treatment group versus the control group as a reference. We additionally adjusted for baseline VAS and ROM, and since an imbalance in the dominant sides affected at baseline was apparent, we also adjusted for this variable in the analysis. For the OSS outcome, the linear regression model was used and adjusted with the baseline OSS score and the number of dominant sides affected.

There was no discernible difference observed between the results obtained from the intention-to-treat analysis, where missing data were imputed using the last observation carried forward method, and the completed case analysis. Consequently, we present the results based on the completed case data analysis.

## Results

### Participants

**Figure 3.**
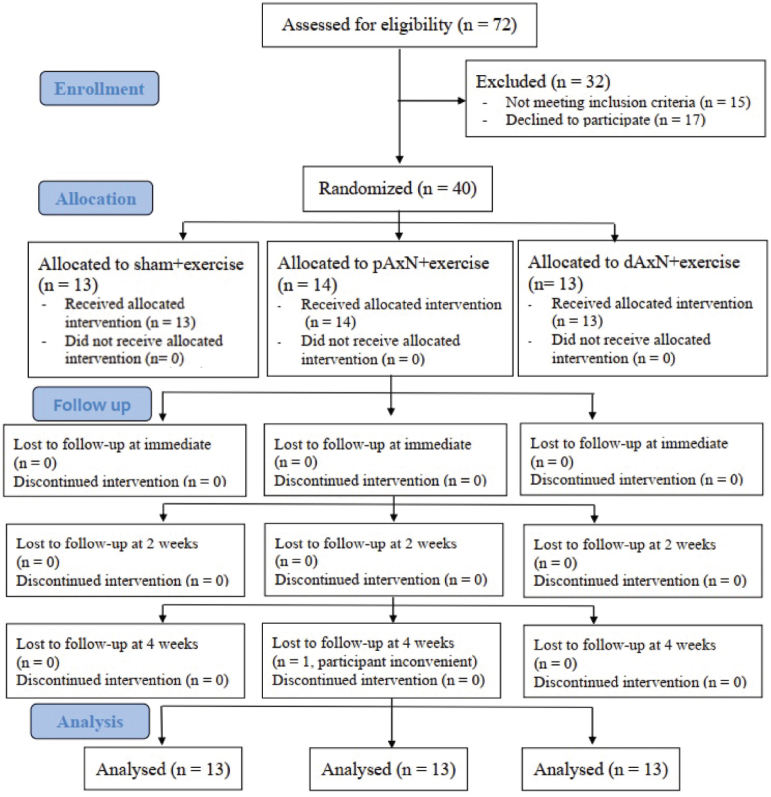
Study flow diagram. dAxN, distal part of the axillary nerve; pAxN, proximal part of the axillary nerve.

Participants recruited from September 2020 to October 2022 included 40 eligible individuals randomly assigned to 3 groups: pAxN (n = 14), sham (n = 13), and dAxN (n = 13). One participant in the pAxN group was lost to follow-up unrelated to the study (**Figure 3**). Baseline characteristics and outcomes of participants are presented in **Table 1**. No serious adverse events occurred during the study period.

**Table 1.** Baseline characteristics of participants

Variables	Treatment groups		
	Control (n = 13)	pAxN (n = 13)	dAxN (n = 13)
**Demographic data**			
Age, mean (SD)	45.77 (11.95)	44.31 (13.17)	45.23 (8.49)
**Sex, n (%)**			
Female	10 (77)	8 (62)	8 (62)
Male	3 (23)	5 (38)	5 (38)
**Duration of shoulder pain, n (%)**			
<6 months	6 (46)	7 (54)	5 (38)
≥6 months	7 (54)	6 (46)	8 (62)
Dominant side affected, n (%)	10 (77)	7 (54)	5 (38)
**Prior treatments, n (%)**			
Medications	4 (31)	5 (38)	5 (38)
Manual exercise	6 (46)	5 (38)	6 (46)
Physical modality	3 (23)	2 (15)	3 (23)
Focus shock wave therapy	1 (8)	0 (0)	1 (8)
Subacromial corticosteroid injection	1 (8)	2 (15)	2 (15)
Dry needling	3 (23)	4 (31)	4 (31)
**Outcomes at baseline**			
VAS, mean (SD)	6.01 (1.56)	6.35 (1.82)	7.29 (1.86)
OSS-TH, mean (SD)	33.15 (3.85)	34.46 (6.98)	32.92 (9.14)
**Active shoulder ROM, mean (SD)**			
Forward flexion	151.92 (15.35)	154.46 (25.52)	143.85 (24.85)
Abduction	137.69 (31.07)	140.00 (43.83)	136.15 (37.03)
Internal rotation	74.62 (19.73)	68.85 (25.18)	73.85 (16.85)
External rotation	76.92 (14.94)	67.69 (28.48)	51.15 (31.70)

1 CI, confidence intervals; OSS-TH, Oxford Shoulder Score Thai version; ROM, range of motion; SD, standard deviation; VAS, visual analog scale.

### VAS

**Figure 4.**
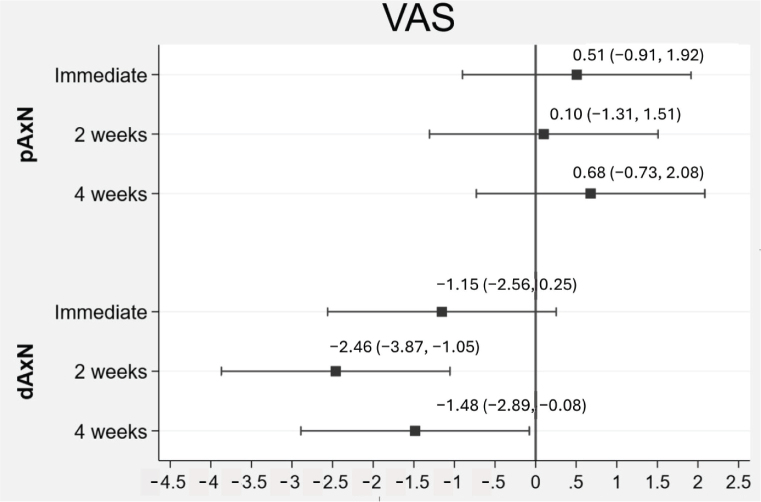
Estimated mean difference of VAS in pAxN and dAxN group compared with the sham group. dAxN, distal axillary nerve; pAxN, proximal axillary nerve; VAS, visual analog scale.

All groups showed significant reductions in VAS scores at 2-weeks and 4-weeks posttreatment. The dAxN group had significantly superior pain reduction compared to the sham group at 2 weeks (−2.46, *P* = 0.001) and 4 weeks (−1.49, *P* = 0.039), with a diminishing effect observed at 4 weeks. However, the pAxN group did not experience a significant pain reduction compared to the sham group (**Figure 4**).

### OSS

While no significant differences were observed between the groups in the OSS outcome at 4 weeks posttreatment, it is worth noting that all groups demonstrated improvement in OSS compared to the initial evaluation (**Table 2**).

**Table 2.** Comparison of the effects of pAxN and dAxN with the sham group immediately posttreatment and at 2- and 4-weeks follow-ups, as well as with in-group comparisons with baseline

Outcomes/time	Study groups			Comparison with the sham group			
	Sham (n = 13) estimated mean (95% Cl)	pAxN (n = 13) estimated mean (95% Cl)	dAxN(n = 13) estimated mean (95%CI)	pAxN – Sham		dAxN – Sham	
				Mean difference (95% Cl)	*P*	Mean difference (95% Cl)	*P*
**VAS** †							
Baseline	6.31 (5.52, 7.09)	6.47 (5.69, 7.24)	6.87 (6.08, 7.66)				
Immediate	4.81 (4.03, 5.60)** P = 0.003	5.48 (4.71,6.26) *P* = 0.052	4.22 (3.43,5.01)*** P < 0.001	0.51 (−0.90,1.92)	0.480	−1.15(2.56,0.25)	0.108
2 weeks	4.42 (3.63,5.21)*** P < 0.001	4.68 (3.91,5.46)*** P < 0.001	2.52 (1.73,3.31)*** P < 0.001	0.10 (−1.31,1.51)	0.889	−2.46 (−3.87,-1.05)”	0.001
4 weeks	2.84 (2.05, 3.62)*** P < 0.001	3.67 (2.90,4.45)*** P < 0.001	1.91 (1.12,2.70)*** P < 0.001	0.68 (−0.73, 2.08)	0.346	−1.49 (−2.89, −0.08)’	0.039
**OSS-TH** ‡							
Baseline	33.17 (30.18, 36.17)	33.83 (30.88, 36.78)	33.54 (30.55,36.53)				
4 weeks	22.94 (19.95,25.94)*** P < 0.001	24.21 (21.26,27.17)*** P < 0.001	24.38 (21.40,27.37)*** P < 0.001	0.62 (−5.28,6.51)	0.836	1.08 (−4.82,6.97)	0.717
**Forward flexion ROM** †							
Baseline	149.97 (144.64,155.30)	150.77 (145.48,156.06)	149.50 (144.17,154.82)				
Immediate	155.35 (150.02,160.68) *P* = 0.140	154.39 (149.09,159.68) *P* = 0.322	150.50 (145.17,155.82) *P* = 0.784	−1.77 (−11.89,8.35)	0.732	−4.39 (−14.50,5.73)	0.396
2 weeks	162.66 (157.33,167.99)** P = 0.001	157.46 (152.17,162.76) *P* = 0.067	156.03 (150.71,161.36) *P* = 0.073	−6.0 (−16.12,4.12)	0.245	−6.15 (−16.27, 3.96)	0.233
4 weeks	166.89 (161.56,172.22)*** P < 0.001	160.92 (155.63,166.22)** P = 0.005	161.03 (155.71,166.36)** P = 0.002	−6.77 (−16.89,3.35)	0.190	−5.39 (−15.50,4.73)	0.297
**Abduction ROM** †							
Baseline	136.72 (126.53,146.91)	138.47 (128.43,148.51)	138.66 (128.51,148.81)				
Immediate	157.10 (146.91,167.30)** P = 0.001	144.62 (134.58,154.67) *P* = 0.332	146.74 (136.59,156.88) *P* = 0.203	−14.23 (−31.83, 3.37)	0.113	−12.31 (−29.91,5.29)	0.171
2 weeks	157.80 (147.61,167.99)** P = 0.001	154.62 (144.58,164.67)* P = 0.011	147.51 (137.36,157.65) *P* = 0.164	−4.92 (−22.52,12.68)	0.584	−12.23 (−29.83, 5.37)	0.173
4 weeks	163.26(153.07,173.45)*** P < 0.001	168.47(158.43,178.51)*** P < 0.001	155.97(145.82,166.11)** P = 0.006	3.46 (−14.14,21.06)	0.700	−9.23 (−26.83,8.37)	0.304
**Internal rotation ROM** †							
Baseline	72.73 (66.84, 78.63)	71.00 (65.14, 76.85)	73.58 (67.69, 79.47)				
Immediate	73.12 (67.22, 79.01) *P* = 0.923	82.15(76.30,88.00)** P = 0.005	80.50 (74.62,86.39) *P* = 0.080	10.77 (−0.19,21.73)	0.054	6.54 (−4.42,17.50)	0.242
2 weeks	69.81 (63.91,75.71) *P* = 0.460	78.69 (72.84,84.54) *P* = 0.052	75.50 (69.62,81.39) *P* = 0.627	10.62 (−0.35,21.58)	0.058	4.85 (−6.12,15.81)	0.386
4 weeks	84.27(78.37,90.17)** P = 0.004	85.23(79.38,91.08)*** P < 0.001	82.81 (76.92,88.70)* P = 0.020	2.69 (−8.27,13.65)	0.630	−2.31 (−13.27,8.65)	0.680
**External rotation ROM** †							
Baseline	66.49 (61.65,71.34)	65.43 (60.65, 70.20)	63.85(59.00,68.71)				
Immediate	68.03 (63.18, 72.87) *P* = 0.638	66.97(62.19,71.74) *P* = 0.638	65.78 (60.92, 70.63) *P* = 0.557	0.00 (−9.07,9.07)	1.000	0.39 (−8.69,9.46)	0.934
2 weeks	71.11 (66.26,75.95) *P* = 0.158	65.04 (60.27,69.82) *P* = 0.906	73.08 (68.23, 77.94)** P = 0.005	−5.00(14.07,4.07)	0.280	4.62 (−4.46,13.69)	0.319
4 weeks	71.26 (66.41,76.11) *P* = 0.145	65.81 (61.03,70.59) *P* = 0.906	70.39 (65.53, 75.25)* P = 0.046	−4.39 (−13.46,4.69)	0.344	1.77 (−7.30,10.84)	0.702

† Estimated means from generalized linear mixed models and adjusted for baseline score and dominant sides affected in VAS and ROM outcomes.

‡ Estimated mean from linear regression model and adjusted for baseline score and dominant sides affected in OSS-TH outcome.

1 *P < 0.05,

1 **P < 0.01, and

1 ***P < 0.001.

1 Cl, confidence intervals; dAxN, distal axillary nerve; OSS-TH, oxford shoulder score Thai version; pAxN, proximal axillary nerve; ROM, range of motion; VAS, visual analog scale.

### ROM

**Figure 5.**
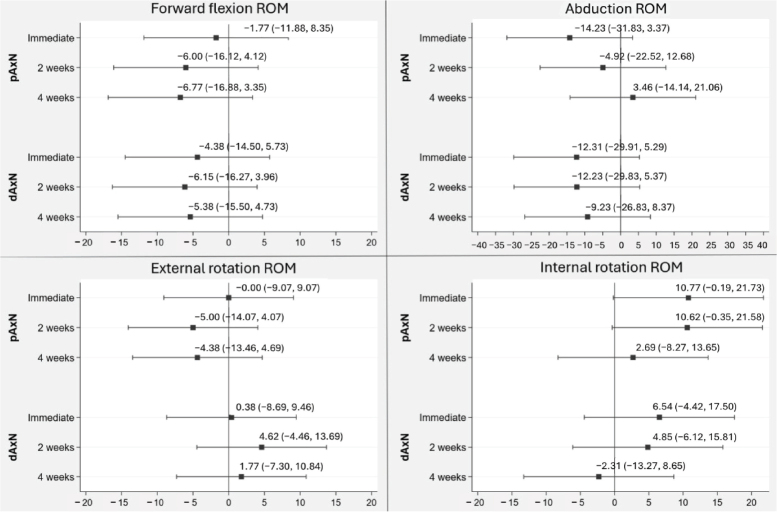
Estimated mean difference of ROM in pAxN and dAxN group compared with the sham group. dAxN, distal axillary nerve; pAxN, proximal axillary nerve; ROM, range of motion.

The active ROM of the shoulder significantly improved in all groups at the 4-weeks evaluation compared to the initial measurements in forward flexion, abduction, and internal rotation. Specifically, the dAxN group showed significant increases in external rotation ROM at 2- and 4-weeks postintervention, but these differences were not significant when compared to the sham group (**Table 2** and **Figure 5**).

### Rescue drug usage

One participant in the sham group was administered a total of 1,000 mg of paracetamol in a single day to alleviate shoulder pain after enrolling in the study. Likewise, a participant from the pAxN group used a total of 500 mg of paracetamol during the study period to relieve shoulder pain. Notably, no participant in the dAxN group reported using paracetamol for pain relief.

## Discussion

The main finding of this study revealed that 5% dextrose PIT administered in the dAxN region significantly alleviated pain in the short term. These findings can be attributed to the anatomical characteristic that dAxN is more specifically associated with cutaneous sensation in the shoulder and arm region compared to the pAxN [23]. Furthermore, compensatory mechanisms for deltoid muscle function are common in cases of rotator cuff tendinopathy [24]. Hence, the effect of 5% dextrose PIT at the dAxN could directly reduce deltoid muscle tone or spasm, leading to a reduction in pain.

Pain reduction after pAxN injection was not significantly different from the sham treatment. This may be due to its lower specificity for shoulder pain, particularly in cases where pain predominantly affects the arm, compared to dAxN. Another possible explanation is the absence of an anesthetic agent in our solution. It is worth noting that injecting a sclerosing substance into deeper anatomical structures could potentially result in postinjection soreness.

Previous studies focused on postoperative nerve blocks were using anesthetic agents for temporally reducing pain [14-16]. To the best of our knowledge, there was no study that investigated the effect of 5% dextrose PIT targeting the axillary nerve. Other than the direct mechanism of 5% dextrose in reducing pain, PIT also involves a hydrodissection effect [25]. By employing fluid to separate nerves from the surrounding soft tissue, this approach aims to alleviate pressure on the “nervi nervorum” that are vital for sensory and motor functions of the peripheral nerve [26]. Compression on the vasa nervorum, which is responsible for blood supply and waste removal from the nerve, can hinder venous outflow and potentially lead to the accumulation of toxins in the affected area. Furthermore, compression can also impact lymphatic drainage, particularly if it occurs outside the epineurium. Consequently, the main objective of hydrodissection is to release the entrapment of peripheral nerves by dissecting and separating them from the surrounding tissues, thereby addressing the underlying pathophysiology.

A previous study determined the minimal clinically important difference in VAS for rotator cuff disease to be 1.40 cm [27]. In our study, the dAxN group demonstrated a statistically significant and clinically meaningful reduction in pain. The estimated mean VAS reduction (95% CI) was −2.46 (−3.87, −1.05) at the 2-weeks follow-up and −1.49 (−2.89, −0.08) at the 4-weeks follow-up, compared to the sham group. To the best of our knowledge, only 1 retrospective study has descriptively reported PIT, in which 2 cases of axillary nerve injection with normal saline for shoulder pain were documented. However, the study did not assess the effectiveness of pain reduction [19]. Most other studies have focused on postoperative pain management, using procedures that involved more than just an axillary nerve block. The results from these studies demonstrated greater pain relief immediately after the nerve block, aiming to reduce analgesic drug intake within the first 72 h postsurgery [14, 17]. However, some unwanted side effects, such as numbness, weakness, or nausea, were reported [14].

Wu et al. have compared PIT using prolotherapy and corticosteroid injections for carpal tunnel syndrome [28]. Their results indicated no significant difference in short-term effects at 1 month. However, the group that received 5% dextrose PIT experienced greater pain relief at 4-6 months. Although our study investigated a different condition—rotator cuff tendinopathy, which does not directly affect the nerve—our findings showed significant short-term pain improvement in the dAxN group compared to exercise alone. This benefit suggests that 5% dextrose PIT may serve as an alternative to corticosteroids, which are associated with more side effects [4, 5].

Beyond PIT, 5% dextrose intratendinous injection has been widely studied in rotator cuff tendinopathy. One study compared 5% dextrose intratendinous injection with corticosteroid subacromial injection and found that while corticosteroids provided better short-term pain relief, there was no difference in pain reduction at 6 months [29]. Using 5% dextrose PIT at the dAxN could potentially fill the gap in short-term pain management, offering an alternative to corticosteroids.

The results of the VAS outcome revealed a decrease in the pain reduction effect after 4 weeks, which is consistent with findings from previous studies [30, 31]. For instance, Lam et al. [30] conducted a study on the analgesic effect of 5% dextrose for deep nerve hydrodissection and reported an average of 88.1% pain reduction during each treatment session. The participants received an average of 3.8 repeated injections over a period of 9.7 ± 7.8 months, suggesting a cumulative effect of pain reduction. Similarly, Li et al. [31] investigated the effect of 5% dextrose PIT in carpal tunnel syndrome and found that repeated injections yielded better outcomes than a single injection. Based on prior evidence [30, 31], the strategy of employing 3 or more injections has been associated with achieving optimal outcomes. However, in this instance, only a single injection was administered in our study, which potentially accounts for the observed transient relief.

Additionally, the temporary pain relief provided by the 5% dextrose PIT at the dAxN could facilitate participation in rehabilitation and physical therapy programs [32]. By alleviating pain, individuals may have increased mobility and ROM, enabling them to engage in exercises and therapeutic activities that are essential for the recovery and rehabilitation of the shoulder [32]. Pain relief during the window period can increase patient compliance with treatment plans. When individuals experience significant pain reduction, they are more likely to adhere to prescribed exercises, medications, and lifestyle modifications, resulting in better treatment outcomes [33]. Furthermore, the window period offers a respite from pain, which can improve mood, reduce stress, and enhance psychological well-being [34].

When evaluating functional outcomes using OSS, we observed a noteworthy enhancement in function across all groups; however, there were no notable differences between the groups. Notably, the improvements seen in each group were deemed clinically significant, as defined by previous studies, which considered changes of more than 6 scores to be significant [35]. These findings align with earlier research indicating that exercise is the primary therapeutic approach for rotator cuff tendinopathy [1, 2].

There was no statistically significant difference observed in the comparison of active shoulder ROM between the groups. However, upon examining the degree change in each plane of ROM, it appeared that the differences did not reach a clinically significant level, defined as a change greater than the measurement error of 15 degrees of motion [36]. Our findings underscore the importance of exercise in enhancing function, reducing disability, and improving shoulder ROM in these patients. Nevertheless, it is crucial to recognize that effective pain management is also essential to promote efficacy and compliance with self-exercise routines.

No serious adverse events were observed during our study, consistent with previous studies [10]. However, it is important to note that this safety profile is contingent upon proficiency and skillfulness in administering ultrasound-guided injections. Furthermore, it is imperative to thoroughly assess the safety profile and potential adverse effects, particularly when dealing with injections into a mixed sensorimotor nerve. Concerns include the possibility of strength deficits in the affected muscles and the occurrence of dysesthesias after the injection. Future studies should explore the long-term effects and overall safety of using 5% dextrose PIT.

Although there is substantial evidence backing the effectiveness of corticosteroid injections for short-term pain reduction, they did not exhibit superiority over prolotherapy in terms of long-term effects [37]. Employing prolotherapy entails fewer substance-related side effects compared to the utilization of corticosteroids [5]. Nonetheless, no prior study has conducted a comparison between PIT targeting the dAxN and corticosteroid injections. Further investigation is necessary to directly assess the comparative efficacy of these 2 treatments in patients with rotator cuff tendinopathy.

Given that our findings demonstrate the safety, affordability, and widespread availability of 5% dextrose PIT at the dAxN, it may serve as a viable alternative or adjunctive treatment option for pain relief in patients with rotator cuff tendinopathy. This approach can be particularly beneficial for patients who have contraindications to conventional pain medications, experience drug interactions, or have hepatorenal impairments. Additionally, it can be considered for patients who have not responded to other conservative treatments, as it opens a window for pain relief and improves compliance with other therapeutic modalities. Nonetheless, it remains crucial to undertake a more extensive clinical trial characterized by adequate statistical potency and extended follow-up durations to validate and confirm our results.

## Conclusion

Combining 5% dextrose PIT at the dAxN with self-exercise demonstrated superior short-term pain reduction compared to self-exercise alone in patients with rotator cuff tendinopathy. This intervention has the potential to serve as an alternative or adjunctive treatment to alleviate pain and enhance the effectiveness of self-exercise. However, further investigation through larger clinical trials is warranted.

We need to address some limitations in our study. First, our sample size was inadequate to allow for subgroup analysis, as we included patients in both the subacute and chronic stages of the disease. Second, in consideration of ethical concerns, we enrolled participants who had not responded to previous treatments, as the use of 5% dextrose PIT for treating rotator cuff tendinopathy in the axillary nerve had not been previously explored.

Further confirmation of our findings is warranted through larger clinical trials or larger cohort studies with longer follow-up periods. It is important to note that the pain-relieving effects of 5% dextrose PIT at the dAxN diminished after 2 weeks. Therefore, additional investigations are needed to assess whether repeated injections could lead to enhanced efficacy in pain reduction.
